# The History of Decompressive Craniectomy in Traumatic Brain Injury

**DOI:** 10.3389/fneur.2019.00458

**Published:** 2019-05-08

**Authors:** Zefferino Rossini, Federico Nicolosi, Angelos G. Kolias, Peter J. Hutchinson, Paolo De Sanctis, Franco Servadei

**Affiliations:** ^1^Division of Neurosurgery, Humanitas Clinical and Research Center, Rozzano, Italy; ^2^Division of Neurosurgery, Department of Clinical Neurosciences, Addenbrooke's Hospital, University of Cambridge, Cambridge, United Kingdom; ^3^NIHR Global Health Research Group on Neurotrauma, University of Cambridge, Cambridge, United Kingdom; ^4^Research Hospital, Humanitas University, Pieve Emanuele, Italy

**Keywords:** decompressive craniectomy, traumatic brain injury, history of head trauma, intracranial hypertension, brain decompression, hemicraniectomy, bifrontal craniectomy

## Abstract

Decompressive craniectomy consists of removal of piece of bone of the skull in order to reduce intracranial pressure. It is an age-old procedure, taking ancient roots from the Egyptians and Romans, passing through the experience of Berengario da Carpi, until Theodore Kocher, who was the first to systematically describe this procedure in traumatic brain injury (TBI). In the last century, many neurosurgeons have reported their experience, using different techniques of decompressive craniectomy following head trauma, with conflicting results. It is thanks to the successes and failures reported by these authors that we are now able to better understand the pathophysiology of brain swelling in head trauma and the role of decompressive craniectomy in mitigating intracranial hypertension and its impact on clinical outcome. Following a historical description, we will describe the steps that led to the conception of the recent randomized clinical trials, which have taught us that decompressive craniectomy is still a last-tier measure, and decisions to recommend it should been made not only according to clinical indications but also after consideration of patients' preferences and quality of life expectations.

## Introduction

Intracranial hypertension is a critical event frequently occurring after traumatic brain injury (TBI) as a delayed secondary pathologic process initiated at the moment of injury. Due to the rigid nature of the skull and the dura, brain edema, expanding hematomas, or blossoming of contusions can rapidly exhaust the compensation mechanisms leading to maintenance of a controlled intracranial pressure (ICP). These events lead to a vicious cycle whereby reduced cerebral perfusion pressure (CPP) causes reduction of cerebral blood flow (CBF) and oxygenation, with worsening of brain edema and, eventually, brain herniation, and death. Following failure of medical management, decompressive craniectomy (DC), a procedure consisting on removal of part of the skull and opening of the underlying dura, can be used as a last-tier therapy to mitigate ICP elevation. During the last century, the popularity of DC has known phases of glory and oblivion, mainly related to alternating surgical outcome, with too many patients suffering severe disability and vegetative state. However, advances in neurointensive care and neuroimaging have led to an increased interest in the use of DC in the 2000s, culminating in the publication of randomized clinical trials (1–3). Despite controversies, the use of DC has been introduced in TBI guidelines, and its efficacy has been recently considered to be beneficial in terms of improving overall survival as a last-tier therapy, compared to medical treatment (4, 5).

We retrace the historical passages which marked the evolution of DC in TBI.

## Early History

### Trephination and Inadvertent Skull Decompression

The earliest evidence of skull trephination dates back to 10,000 BC at the beginning of the Neolithic period and has been deduced by studying the major skull collections: the French Prunières collection and the Peruvian skulls (6). There is limited archeological evidence of trephined skulls found in Egypt, except for few cases analyzed by Pahl in the book Altägyptische Schädelchirurgie (7).

Later, the practice was well-described in the Greek Era by Hippocrates (8). In Alexandrian school, the main records in head injured patients come from the scientist Aulus Aurelius Cornelius Celsus (25 BC–AD 50). He advocated trephination when patients developed symptoms after trauma despite the absence of any fracture. In the 2nd century AD, during the Roman Empire Era, Galen suggested trephination for depressed fractures, fractures with hematoma, comminuted fractures, and trichiasis (superficial gouging of the bone). In the Early Medieval Period, the increasing recognition of importance of anatomic barrier provided by skull and dura, lead to a decline in popularity of cranial surgeries. Despite this tendency, several examples of medieval neurosurgical skills have been demonstrated by archeological findings, originating from area of Italy and Hungary and dated for early to mid-middle ages (9–12). However, very little knowledge was added to the neurosurgical management of cranial injuries until the medical school in Salerno, Italy, regenerated interest in cranial surgery in the 11th century (13).

## The Masters

### Berengario da Carpi (14)

**Figure 1:**
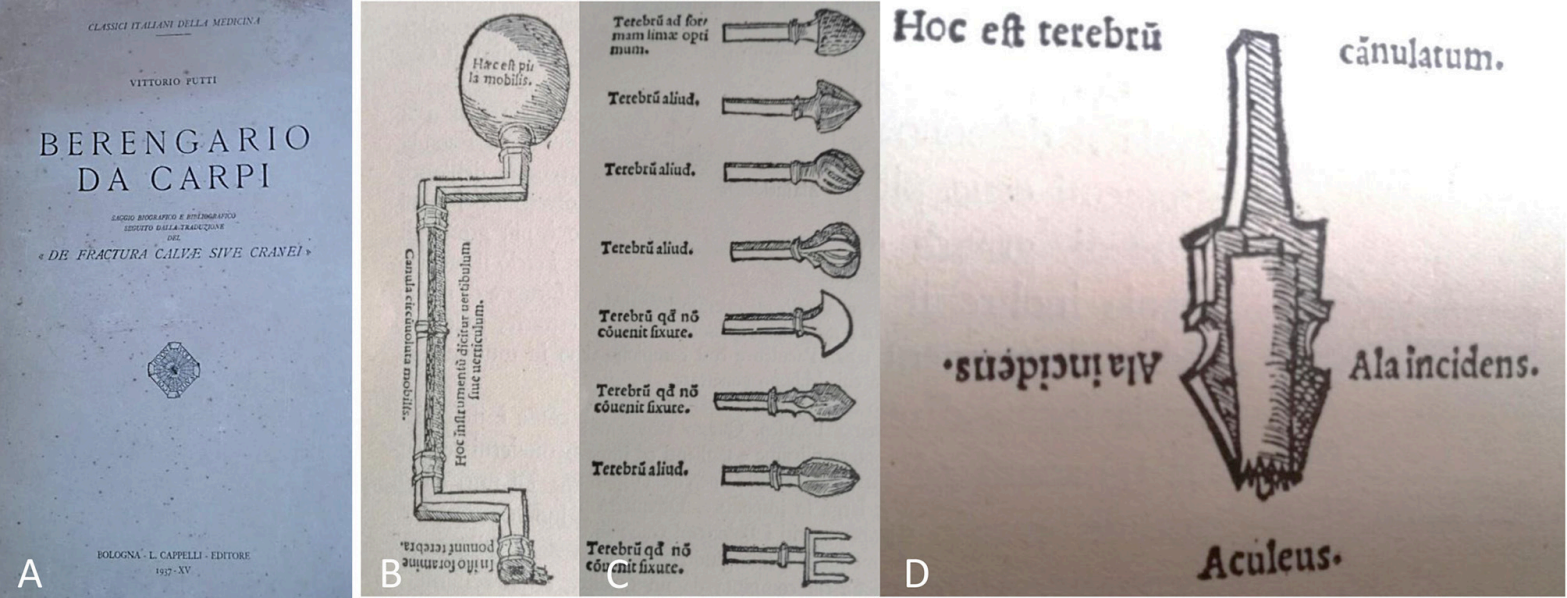
**(A)** Frontispiece of De Fractura Calvae sive Cranei, original Italian translated copy (from *Vittorio Putti, Berengario Da Carpi “De Fractura Calvae sive Cranei”, Bologna—L. Cappelli Editore, 1937, private collection*. Figure is in public domain and no permission is required for reuse). **(B–D)** These pictures show some of the surgical instruments in use at that time to perform a trephination.

Berengario da Carpi was an Italian physician and teacher of Anatomy at the Bologna University. After taking care of Lorenzo de' Medici, suffering from an occipital gunshot wound, he was inspired to write in 1518 “Tractatus de fractura calve sive cranei” (10). To our knowledge, the manuscript contains the first description of indications and technique of craniotomy. He reported three cases of brain injury successfully operated on, with 1 year follow up. One of these patients underwent also DC. He also reported a detailed description of surgical instruments and of the costs of the various procedures (Figure 1) (15).

## EugÈne-Louis Doyen (1859–1916): the Temporary Hemicraniectomy

**Figure 2:**
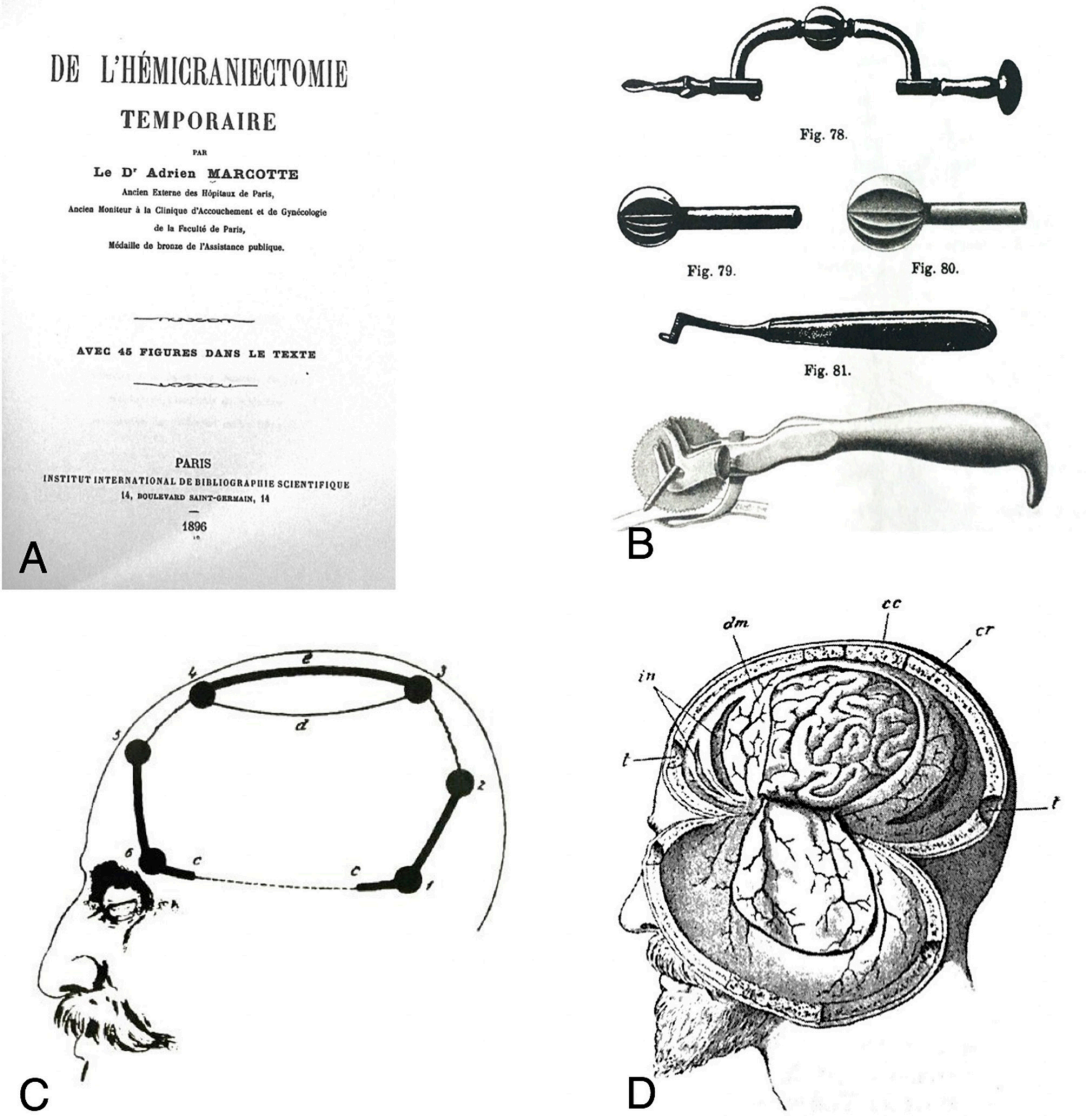
**(A)** Frontispice of De l'hémicrniectomie temporaire, by Charles Adrien Marcotte. **(B)** Sample of the surgical instruments used by Doyen. **(C)** Lines and burr holes showing the extension of the temporary craniectomy. **(D)** Intradural view after performing temporary hemicraniectomy: the dural flap is usually downward overturned [from Marcotte (16). Figure is in public domain and no permission is required for reuse].

The first scientific reference and description of an hemicraniectomy was reported in 1896 by Charles Adrien Marcotte in his graduation thesis in Medicine and Surgery, named *De L'hemicraniectomie Temporaire* (16). The innovation of the *hemicraniectomie temporaire* consists of the realization of a large fronto-temporo-parietal bone flap (*volet osseux*), with the bone left adherent to periosteum, temporal muscle, subcutaneous tissues, and skin. The adhesion of the bone flap to the soft tissue would have limited wound defects, bone resorption and loss of substance (Figure 2) Although it was not used to treat severe TBI, the power of this technique in lowering increased intracranial pressure (i.e., in cases of meningitis) had already been introduced by Marcotte.

DC was described by Annandale in 1894 as a palliative procedure for inoperable brain tumors (17). Nevertheless, the most relevant experiences on DC in head trauma took place in the XX century.

### Kocher and Cushing

**Figure 3:**
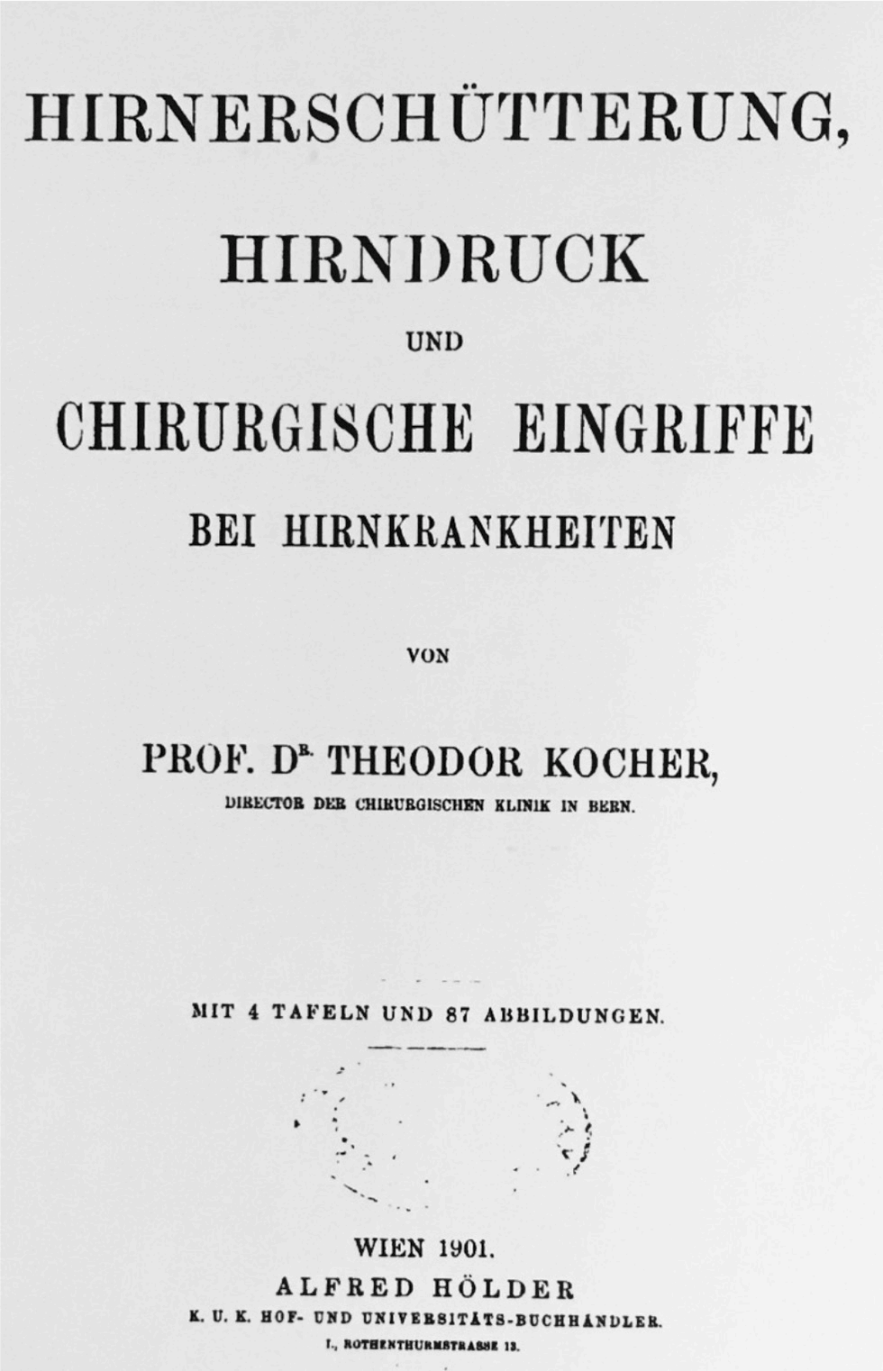
Frontispiece of the manuscript by Dr. Theodor Kocher [from Kocher (18). Figure is in public domain and no permission is required for reuse].

The use of “large” DC for patients with raised intracranial pressure following TBI was firstly reported by Kocher in 1901. In his manuscript (Figure 3), he makes a systematic study of brain trauma and CSF circulation, and reported the therapeutic measures to be adopted in order to manage intracranial hypertension. In the Chapter VIII, he advocates the use of trephination, as soon as possible, in all cases of intracranial hypertension. In the Chapter XVIII he suggests to perform the temporary hemicraniectomy in selected cases where a pressure relief cannot be achieved by trephination alone (18).

From the lesson learned watching Kocher in Bern, US-neurosurgeon Cushing proposed DC for the treatment of other brain disorders (19–21).

**Figure 4:**
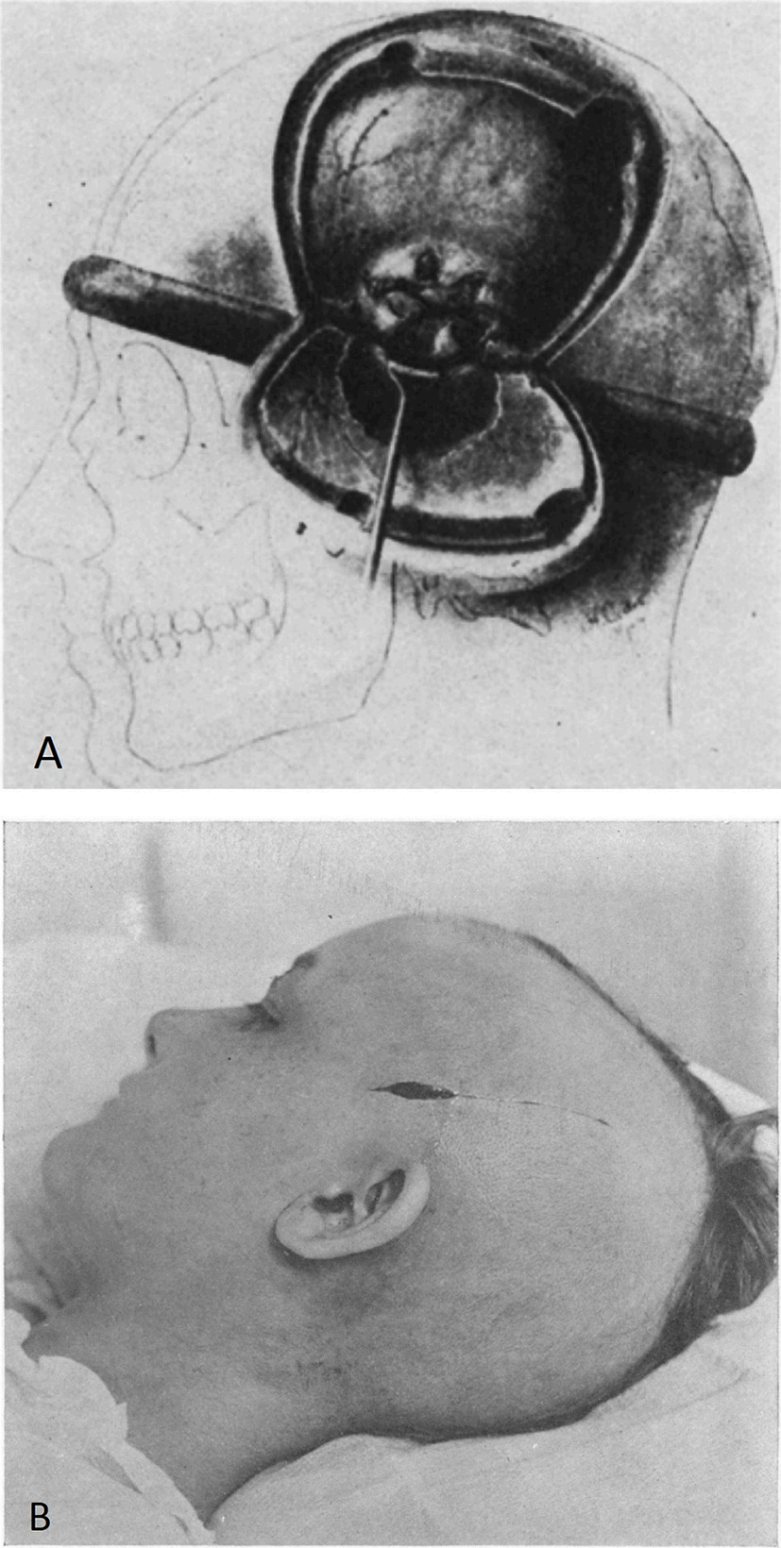
**(A)** Decompressive measures described by Cushing for the management of cerebral hernia in inaccessible brain tumors [from Cushing (21). Figure is in public domain and no permission is required for reuse]. **(B)** Incision of the scalp for subtemporal craniectomy [from Cushing (20). Figure is in public domain and no permission is required for reuse].

In 1905, he reported the use of DC for inaccessible brain tumors (Figure 4A).

Only in 1908, he described the subtemporal DC for the intracranial complications associated with bursting fractures of the skull (20). The subtemporal craniectomy technique consisted of a linear incision of scalp, splitting of the fibers of the temporal muscle and a 4.5 cm diameter bone removal with dural opening (Figure 4B).

The immediate reduction of intracranial pressure had a favorable impact in reducing morbidity in survivors, compared with patients who did not undergo surgery (19, 20).

The indication by Cushing for decompressive craniectomy with aggressive wound debridement of fragments in penetrating brain injury followed his observation of 250 cases in War World I (22). The same recommendation was later supported by Matson, after analyzing World War II and Korean War head trauma data, and continued during the conflict in Vietnam (23). Cushing advocated watertight dural closure, a principle less valid in wartime nowadays. However, the DC in wartime goes beyond the scope of this paper and has been properly described elsewhere (24).

### Hemicraniectomy, Bifrontal, and Subtemporal Craniectomy

After the preliminary experiences, clinical practice showed poor clinical outcomes. Therefore, DC quickly felt into discredit. From 1960 to 1980, only twenty-two papers dealing with DC in TBI were published, with a mean mortality rate from 46 to 96%, regardless of the surgical technique used (17, 25–34).

Two main techniques would have represented the standard during the next years.

The bifrontal craniectomy, reported by Kjellberg and Prieto in 1971, was performed in 50 patients with TBI. The main passages of the surgical technique are as follows: “*The reference points for the bone flap are: a burr hole over the frontal sinus; burr holes in the zygomatic portion of the frontal bone at the anterior insertion of the temporalis muscle; a burr hole 1 cm posterior to the coronal suture in the midline; and two burr holes laterally in the temporal region near the coronal plane of the midline burr hole. The burr holes are connected by a saw and the frontal bone removed, ordinarily in two halves. The dura is…incised bilaterally above the supraorbital ridges to the sagittal sinus anteriorly…The sinus and falx are divided by scissors”*

Kjellberg and Prieto did not think that this procedure was simply prolonging the life of patients with irreversible damage, but with proper indication could result in reasonable outcomes. They deplored its application in patients with modest injury, and noticed that younger survivors, even if they had a decerebrate state at presentation, had a better potential for good neurological recovery than the adults. They suggested “*the following indications as a guide to the decision to use this procedure: 1. Coma: totally unresponsive or responsive only to deep pain 2. Unilaterally or bilaterally dilated and fixed pupils 3. Apnea 4. Decerebrate posturing…at least two of the indications above should be present.”* (30). In 1975, Venes and Collins made a retrospective analysis of 13 patients who underwent primary bifrontal DC for the management of post-traumatic cerebral edema. They reported a significant decrease in expected mortality (30.8%), but severe morbidity in the survivors, and only one 2 years-old patient completely recovered (34).

During the same year, Gerl and Tavan reported that extensive bilateral craniectomy with opening of the dura offers the possibility of rapid reduction of intracranial pressure. However, they observed 70% of mortality, and only 20% of the cases with full recovery (28).

The second technique is the evolution of the hemicraniectomy and would have represented the most popular mean of DC for several years. Ransohoff et al. reported their experience in thirty-five patients with “*unilateral acute subdural hematoma associated with predominantly unilateral underlying cerebral contusions and lacerations*.” The authors referred a survival rate of 35%, with 7 patients returned to their normal occupation. According to these findings, hemicraniectomy seemed to show favorable results in patient with malignant cerebral edema, compared with previous series (33). The technique of hemicraniectomy by Ransohoff is described as follows: “…*a skin flap was extended from the glabella along the midline, terminating 4 cm above the external occipital protuberance. The skin incision was carried laterally to the level of the transverse sinus, and a one-layer skin flap including the periosteum was turned. A frontoparietal, occipital, and temporal bone flap was then removed to reveal almost the entire surface of the hemisphere… The temporal squama was rongeured to the floor of the temporal fossa, with the neurosurgeon making absolutely certain that no shelf of bone remained that might prevent subsequent lateral shift of swollen temporal lobe. The bone flap was discarded or placed in the bone bank. The dura was widely opened and hinged at the attachment of the superior sagittal sinus. Through this exposure it was possible to carry out a complete removal of all solid and liquid hematoma. The inferior surfaces of the frontal and temporal lobes were inspected for areas of clot and contusion….Bleeding from brain lacerations was controlled, and badly macerated brain was resected, if necessary. The bridging veins along the sagittal and transverse sinuses were inspected for active bleeding and were often found to be the source of the subdural hematoma. When hemostasis was satisfactory, the dura was laid over the surface of the brain, with no attempt at closure. …The scalp was closed in a one-layer on-end mattress technique….”*

The favorable effects of hemicraniectomy on limiting intracranial hypertension were also found in 1973 by Morantz et al. as well. The authors analyzed the radiological modification of midline shift in eleven patients with subdural or epidural hematoma underwent DC. In arteriograms, “*there was a general correlation between the degree of postoperative shift and the clinical status of the patient; the patients showing the best response displayed the least displacement of the midline structures and vice-versa.”* (31).

## The End of the Story?

In 1976, the experience of Cooper et al. seemed to establish the end of DC as a standard practice to limit the intracranial hypertension linked to the cerebral edema. He reported a 10% total and a 4% functional survival rate in 50 patients with TBI. No correlation with survival and patient's age, status of preoperative neurologic examination, angiographic findings, and appearance of the brain at operation was found (17, 35).

However, Cooper et al. recognized the value of DC only as a second tier treatment in deteriorating patients with no brainstem dysfunctions:

“*The operation of hemicraniectomy should be restricted to those patients who enter hospital, obtunded but without demonstrable brain stem dysfunction, only to deteriorate subsequently because of increasing hemispheric edema and/or subdural clot*” (17).

## The Dark Age of DC

Despite the unfavorable results discouraged further investigations, some groups, particularly in Japan, continued to carry on research about the role of DC in TBI (36–38).

In 1979, Yamaura and Makino analyzed the effects of DC in patients with cerebral contusion. The authors stratified patients in different groups according to their age and the pre-operative clinical status (*key signs*: pupillary changes, decerebration and respiratory disturbance). Their findings were not different from previous studies: mortality rate was 23% in 0–29 vs. 40% in >30 years-old patients, and >30 years-old patients had poor functional recovery. Mortality was therefore lower in younger patients (36).

During the same years, Shigemori et al. published a short series of 15 patients with SDH treated with DC. Despite a poor post-operative outcome, the authors reported that the midline shift and the ICP were not significantly modified in all patients with severe brain swelling, but mainly in the subgroup of patients with mild elevated intraoperative ICP (37).

However, some questions remained pending: (1) does the time from the traumatic event impact on mortality rate? (2) which is the pre-operative ICP value as a cut off for surgery and how does it relate to a favorable outcome? (3) Does pre-operative clinical status affect the post-operative outcome?

In 1980, Shishido et al. found that patients with lower ICP (10–30 mmHg) who underwent DC had a better post-operative neurologic status compared to patient with rapidly increasing post-operative ICP or with higher values (40–70 mmHg). This study showed how the ICP seemed to be a crucial element able to influence the response to therapy in patients with TBI and diffuse cerebral damage (38).

## The Rebirth of DC

The improvement of ICP monitoring techniques and the widespread adoption of therapies to reduce intracranial pressure, i.e., mannitol, hyperventilation, barbiturates, extended the care of post-traumatic intracranial hypertension to a multidisciplinary team, mainly composed by surgeons and neurointensivists. Indeed, it allowed to reduce the application of DC only to selected cases, with brain edema not responsive to medical treatment, as a second-tier therapy (39–44).

Moreover, the reported success of DC for stroke (45, 46) was also a factor contributing in renewing interest in DC for TBI.

According to this, in 1988 Gower et al. proposed a step-by-step treatment algorithm for patients with closed head injury. The authors examined 115 patients with severe closed head injury, with invasive monitoring of ICP, started on a regimen of medical treatment (head elevation, fluid restriction, chemoparalysis, hyperventilation at PCO2 25-30 torr and, if not responsive, mannitol). ICP above 20 mmHg triggered further therapeutic maneuvers including skull decompression. In the group of decompressed patients, 40% survived, compared with 82.4% of patients in pentobarbital coma group without decompression. Some important information came from this study: (1) the treatment of intracranial hypertension had to be guided by the ICP value; (2) the DC could be efficacious as second-tier therapy; (3) however, the mortality rate in the decompressed group was not changed yet if compared to the past (40).

In 1990, Gaab et al. with a prospective study design treated 37 patients <40 years old. They performed 19 bifrontal craniotomies and 18 hemicranietomies, and reported 5 deaths (13.5%), 3 vegetative states (8.1%), while all other patients achieved full social rehabilitation or remained moderately disabled; they established as best predictor of a favorable outcome an initial posttraumatic Glasgow coma scale (GCS) ≥7 (37).

Another interesting observation was described by Yamakami and Yamaura (44). They observed a significant relationship between the increasing of CBF, assessed by SPECT99m technetium-hexamethyl-propyleneamine oxime, recorded 24 h after DC, and an improvement of GCS score (40).

Between the end of 1990s and the first years of 20th century, some authors (47–52) tried to establish a new role for surgical bone flap decompression and duraplasty in the treatment of severe head injuries.

Polin et al. confirmed that timing had a positive impact on ICP control. Furthermore, pre-operative higher GCS (≥6) and younger age were positive predictor of good outcome (50).

In 1999, Guerra et al. conducted a prospective clinical study on the effect of bilateral or front temporal craniectomy in patient with refractory intracranial hypertension not responsive to medical therapy. Their results looked surprisingly good: only 11 patients (19%) died; five patients (9%) survived, but remained in a persistent vegetative state; six patients (11%) survived with a severe permanent neurological deficit, and 33 patients (58%) attained useful social rehabilitation. According to them, DC was indicated in patients <50 years-old, with brain swelling on CT scan, no fatal primary brain injury, before irreversible brainstem damage or generalized ischemic brain damage (monitoring of ICP, and B wave, AEPs, & SEPs) had occurred (48).

In 2000, Munch et al. assessed how unilateral DC could modify ICP, CPP, and few CT parameters like brain shift and status of the mesencephalic cisterns. DC was performed as primary-tier therapy in 63.3% and as secondary-tier therapy in 36.7% patients. Despite a significant reduction of midline shift, this finding did not correlate with a better patient outcome, that was favorable in only 41% patients (49). Differently from the results by Polin, timing seemed not to be related to patient's outcome, as confirmed by Whitfield and Guazzo (52).

Thanks to these authors, we understood that DC was effective in improving brain elasticity, reducing ICP, improving CBF and overall survival, but not the functional status.

In summary, at the end of the 20th century, the indications for DC were the following: ICP >30–35 mmHg or CPP <45–70 mmHg, age <50 years, GCS>4, CT signs of brain swelling, associated masses, GCS 3 plus bilateral fixed pupils excluded (48, 50–52). Two conditions for DC were already indicated even if not well-defined yet: primary, if associated with haematoma evacuation (49); secondary, if followed ICP increase not treatable with medical therapy (48, 50–52).

The main conclusions drawn from the few studies dealing with the role of DC in post-traumatic diffuse brain injury were: (1) decompression had to be performed in selected cases, mainly young patients with GCS not inferior 7 and without signs of irreversible brain damage, only after failure of intensive medical care; (2) timing, age and post-operative ICP could have a significant impact on post-operative outcome; (3) the therapy had to be focused on maintaining a stable ICP (<20 mmHg); (4) despite the surgical and anesthesiological advances, the outcome of operated patients did not substantially improve. The number of patients with a good recovery or a moderate disability was still about 30%.

However, at that time no randomized controlled trials had been still carried on.

## The Era of Randomized Trials

During the 21st century, DC in TBI has become very popular again, with a striking increase in the number of published papers.

Most of these papers are single or multi-center retrospective series, case reports and reviews (53–58).

Until now, three randomized controlled trial (RCT) have been carried on and one (RESCUE-ASDH trial) is ongoing. The trials differ in terms of study population: inclusion criteria, methods and outcome (Table 1), (1–3) and criticisms have been raised, for example in terms of the inclusion criteria for the DECRA trial (60–63). Kolias et al. have recently compared and discussed the DECRA and RESCUEicp trials (59).

**Table 1:** Differences between the RCTs by Taylor et al. (2) DECRA and RESCUEicp trials.

	**Taylor et al. (2)**	**DECRA**	**RESCUE-icp**
Recruitment up to 72 h post-TBI	100%	100% of patients	56% of patients
TBI type	Diffuse injury and/or mass lesions	Diffuse injury only	Diffuse injury and/or mass lesions (including contusions and evacuated hematomas)
ICP threshold	ICP 20–24 mmHg for 30 min, 25–29 mmHg for 10 min, 30 mmHg or more for 1 min	> 20 mmHg for 15 min in 1 h	> 25 mmHg for at least 1 h
ICP-lowering therapies before randomization	Tier 1	Tier 1	Tiers 1 and 2
Pooled mortality	33.30%	18.7%	37.5%
Mortality in DC vs. medical group	11.1 vs. 22.2%	19 vs. 18%	26.9 vs. 48.9%
Documented follow-up	6 months	6 months	6 and 12 months
Poor outcome (medical group vs. surgical group)^*^	86 vs. 46 %, *p* = 0.046^∧^	51 vs. 70%, *p* < 0.01	65.4 vs. 57.2%, *p* = NS (6 months)
			67.7 vs. 54.6%, *p* < 0.01 (12 months)

*From Kolias et al. (59) used and modified with permission*.

∧ *The modified Glasgow Outcome Score (GOS) to obtain a functional outcome*.

* *In the DECRA trial, the upper sever disability (patient independent only at home) was considered among the poor outcomes, in the RESCUEicp trial, in view of the indication to surgery as last tier, it was considered as good outcome*.

In conclusion, current evidences from multicenter clinical trials suggests that early neuroprotective bifrontal DC for mild to moderate intracranial hypertension is not superior to medical management for patients with diffuse TBI. DC used as a last-tier therapy for patients with severe, sustained, and refractory posttraumatic intracranial hypertension leads to a substantial mortality reduction but increases disability compared to medical management. However, at 12 months there was a significant difference in the number of patients with a favorable outcome (defined as upper severe disability—independent at home for at least 8 h) compared to the medical management (3, 64, 65).

## Lessons From the Past: *Errare Humanum Est Perseverare Autem Diabolicum (To Make Mistakes Is Acceptable, But not to Repeat Them…*)

The technique of DC as a therapy to reduce ICP has ancient roots. We have learned from the past that DC is an extreme measure, not a panacea for any case of increased ICP. Indeed, a significant percentage of survivors have moderate to severe neurological sequelae. Therefore, decisions to recommend DCs must always be made not only in the context of “*its clinical indications but also after consideration of an individual patient's preferences and quality of life expectation”* (66).

## Author Contributions

All the authors meet the 4 criteria according to the ICMJE (International Committee of Medical of Medical Journal Editors). In detail, ZR and FS had a substantial role in designing and drafting the paper. AK and PH significantly contributed to the analysis, interpretation critically revising the work. FN and PD equally contributed to the acquisition and interpretation of data.

### Conflict of Interest Statement

FS received limited educational grants and consultancy fees from Finceramica, Integra and Takeda company totally unrelated to the content of this paper. The remaining authors declare that the research was conducted in the absence of any commercial or financial relationships that could be construed as a potential conflict of interest.
